# Supplementary figures and images for: rRNA transcription is integral to phase separation and maintenance of nucleolar structure
Source: PLoS Genet. 2023 Aug 28;19(8):e1010854. doi: 10.1371/journal.pgen.1010854 (PMC10513380; doi:10.1371/journal.pgen.1010854)

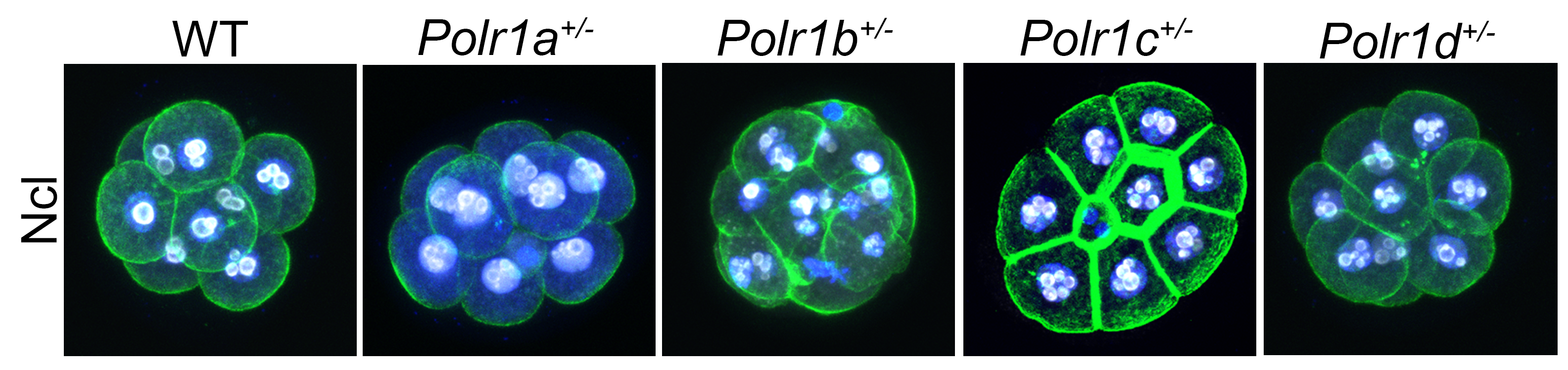

Supplement: S1 Fig — Immunostaining with Ncl to visualize NPBs indicates that its expression levels are consistent, and the number of NPBs per blastomere are similar between WT and Pol I heterozygous mutants. Scale bar = 12.5 μm. (TIF) [file pgen.1010854.s001.tif]

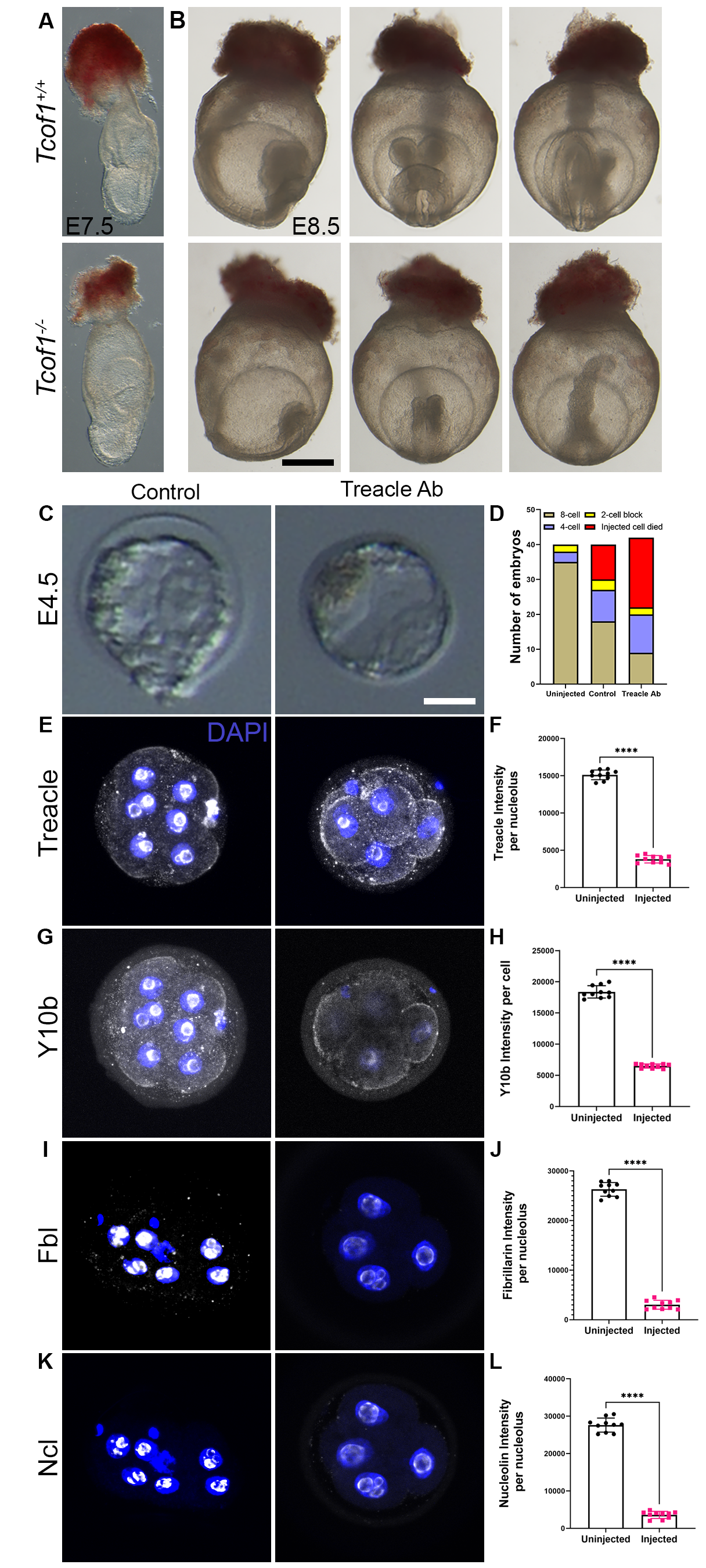

Supplement: S2 Fig — (A) Tcof1-/- embryos are noticeably smaller than controls at E7.5. At E8.5 (B), these embryos are significantly smaller than their WT littermates. (C) Treacle antibody injected embryos exhibit cell death in the inner cell mass and fail to hatch, while control embryos proceed to hatching at E4.5 (D) Survival statistics of embryos injected with antibody indicates that over 50% of the embryos injected with Treacle antibody did not survive. Expression levels of Treacle (E, F), Y10b (G, H), Fbl (I, J) and Ncl (K, L) are significantly reduced in Treacle injected blastomeres and their descendants. The data is represented as mean+/-SEM. Scale bar for A and B is 100 μm. Scale bar for E, G, I and K = 12.5 μm. **** indicates p<0.0001. (TIF) [file pgen.1010854.s002.tif]

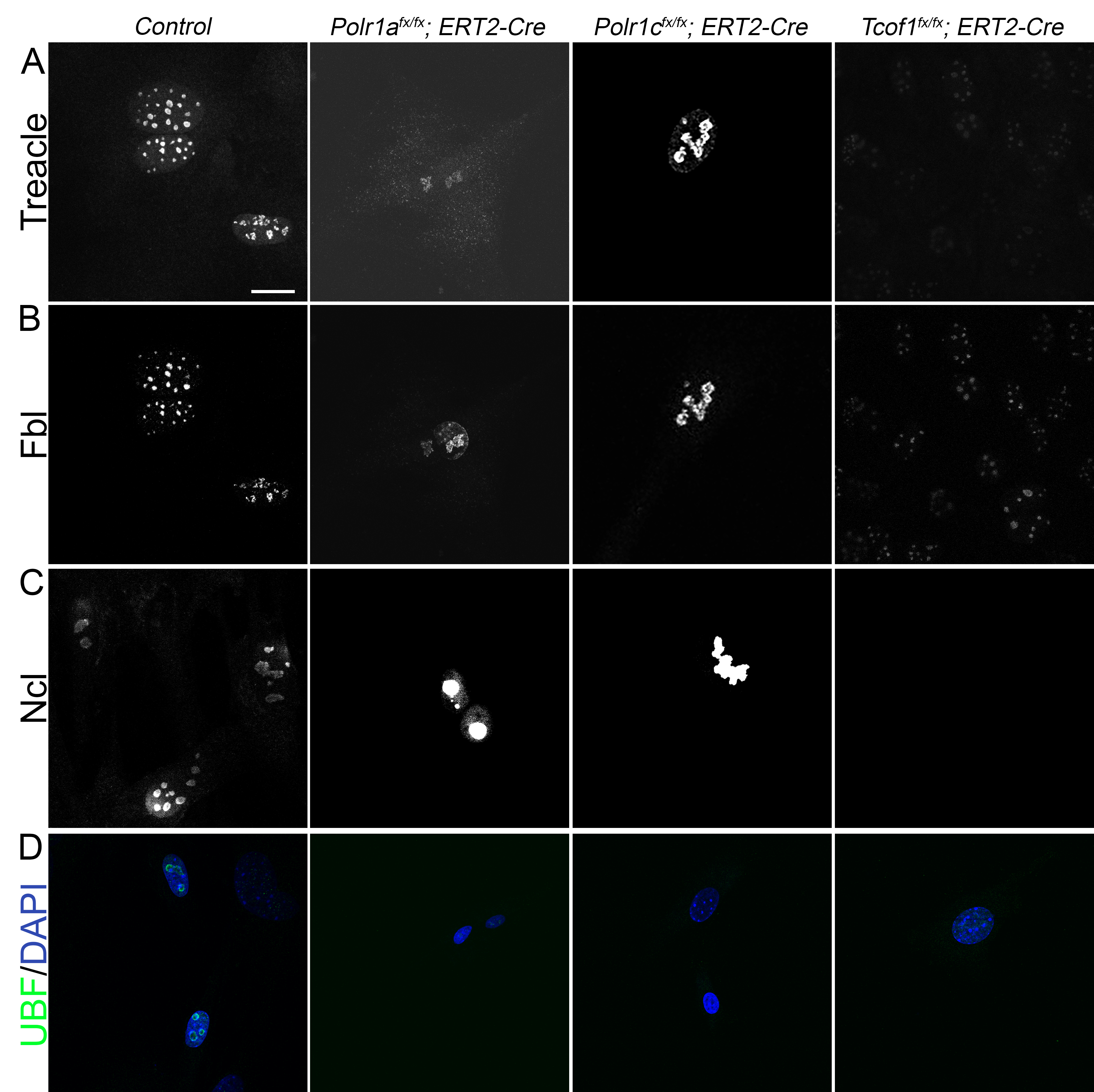

Supplement: S3 Fig — Immunostaining of MEFs with Treacle (A), Fbl (B), Ncl (C) and UBF (D) antibodies suggests that the number of nucleoli are significantly reduced in Polr1afx/fx;Cre-ERT2, Polr1cfx/fx;Cre-ERT2 and Tcof1fx/fx;Cre-ERT2. Tcof1fx/fx;Cre-ERT2 MEFs have reduced expression of Fbl, Ncl and Ubf unlike Polr1afx/fx;Cre-ERT2 and Polr1cfx/fx;Cre-ERT2, compared to controls. (TIF) [file pgen.1010854.s003.tif]

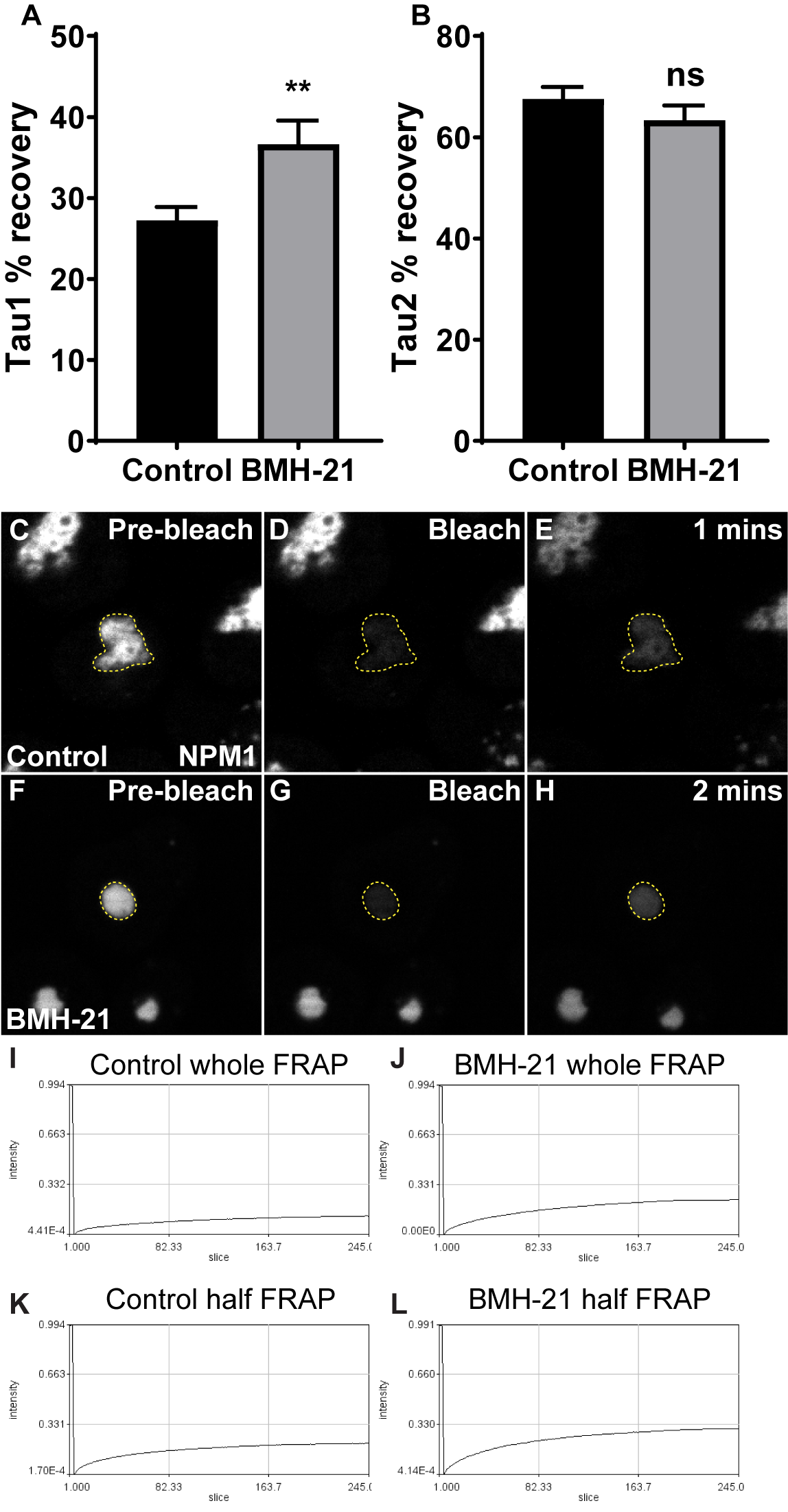

Supplement: S4 Fig — (A, B) Graphs showing the percent recovery of the GC of half and whole FRAP respectively. Data is represented as mean+/-SD. Student’s t-test. (C-H) Confocal images of whole FRAP NPM1 and its recovery over time in control and BMH-21 treated cells. (I-L) Average FRAP recovery curves for control (I, K) or BMH-21 treated (J, L) cells. The x-axis indicates slices or frames of the video where each slice or frame is the image taken at 0.5s intervals and the y-axis displays the intensity of the recovering NPM1 in the GC. ** indicates p<0.01, ns indicates p>0.05. (TIF) [file pgen.1010854.s004.tif]

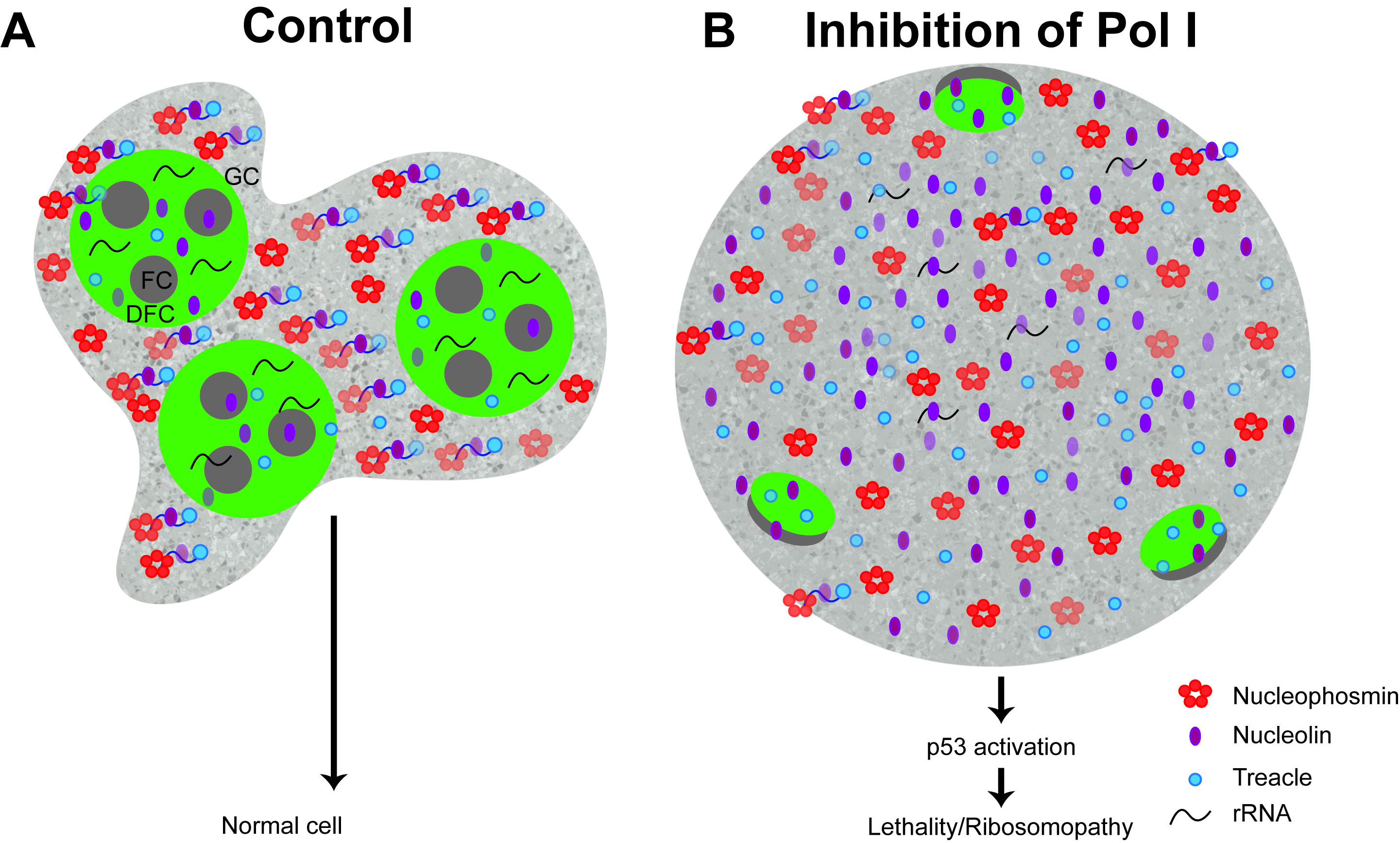

Supplement: S5 Fig — (A) In control embryos and cells, each nucleolus has distinct FC and DFC regions surrounded by the GC and maintains an amorphous structure. rRNA is bound to nucleolar proteins (NPM1 in red, Ncl in purple and Treacle in blue). (B) When Pol I activity is inhibited, the nucleolus changes shape, becoming round and condensed. rRNA transcripts are reduced and nucleolar protein expression increases, which leads to a change in phase separation of the nucleolus. This leads to nucleolar stress in the pathogenesis of ribosomopathies. (TIF) [file pgen.1010854.s005.tif]
