# Supplementary material for: rRNA transcription is integral to phase separation and maintenance of nucleolar structure
Source: PLoS Genet. 2023 Aug 28;19(8):e1010854. doi: 10.1371/journal.pgen.1010854 (PMC10513380; doi:10.1371/journal.pgen.1010854)
Supplement: Attachment — Submitted filename: Response to Reviewers Final.docx [file pgen.1010854.s009.docx]

**Response to the editors:**

The manuscript was fully evaluated at the editorial level and by two independent peer reviewers, who differed in their enthusiasm for the work. Reviewer #1 had a number of serious concerns regarding interpretation of some of the cell biologic and molecular events, and recommended rejection; reviewer #2 had several minor concerns that could be addressed with additional discussion.

The manuscript and the reviews have now been discussed among members of the editorial board. While we appreciate and understand the concerns raised by reviewer #1, there is a consensus that the multiple mutant in vivo and hiPSC analyses represent an important contribution for the genetics community. Regarding specific comments from reviewer #1, we do not think direct measurement of rRNA synthesis or steady-state levels of Pol1 subunits (comment #4) are necessary, nor do we think additional mutant studies, e.g. of PolR1F (comment #3), are needed. However, we do ask that you discuss the concerns raised by reviewer #1 regarding the relationship of nucleolar "remnants" described in your work to previous work on cell biology of the nucleolus (comments #1, #3). We also ask that you discuss additional concerns raised by reviewer #1 in confidential comments to the editor regarding the rigor of the conclusions regarding phase separation, and what additional studies might shed light on this question in the future.

We therefore ask you to modify the manuscript according to the review recommendations. Your revisions should address the specific points made by each reviewer.

- We sincerely thank the editors for recognizing the importance of our work and for their editorial judgement and recommendations regarding which reviewer raised issues needed to be addressed.
- We were not able to access any confidential comments to the editor regarding the rigor of the conclusions regarding phase separation. However, to ensure we are transparent about the rigor and reproducibility of our experiments we provided additional details in the methods section regarding the FRAP experiments, detailing the number of experiments performed and number of cells analyzed per experiment.

**Response to reviewers:**

We sincerely thank the reviewers for their thorough and insightful critiques of our paper, and their recognition of the importance of our work. We have revised our manuscript accordingly to address the comments and suggestions from the reviewers as described below. Changes to the text are highlighted for ease of reference. We trust the reviewers will now find the work satisfactorily addresses their concerns and is suitable for publication.

Reviewer #1: This manuscript demonstrates the requirement for rRNA transcription during development. Its particular value is the demonstration that the knockout of RNA polymerase I and III subunits results in nucleolar defects. While it is assumed that the lack of the polymerase subunits results in the inhibition of rDNA transcription and that the inhibition of embryo survival is due to the inhibition of rRNA synthesis. However, the authors do not measure rRNA synthesis. The Y10b antibody measures the steady state of the intermediates in the nucleolus and not their synthesis.

- We agree with the reviewer that the steady state levels of rRNA transcription data would be valuable to the field, and that we do not measure rRNA synthesis directly in our Pol I mouse mutant pre-implantation embryos. However, we recently showed in neural crest cell (NCC) specific knockouts of *Polr1a*, *Polr1c* and *Tcof1*, that rRNA transcription as measured by qPCR of the 5’ ETS region of 47S rRNA is specifically reduced in NCC in post-implantation embryos in response to Pol I subunit and associated factor loss-of-function (Falcon et al., 2022, PNAS). We have also noted the correlation between Pol I subunit loss-of-function and downregulation of rRNA transcription as measured by 5’ETS qPCR in our zebrafish models of perturbed Pol I function (Weaver et al 2015; Noack Watt et al 2016; Watt 2018).
- However, we cannot perform the same qPCR experiment in the pre-implantation embryos because a pre-implantation embryo can only be used either for a DNA or RNA isolation experiment. DNA isolation was necessary for genotyping the embryo to identify the wildtype and the mutant embryos. For a qPCR experiment, at least 20 embryos each of the wildtype and mutants would be required which would be impossible to pool without genotyping.
- The reviewer is correct that Y10b does not measure direct rRNA synthesis, and we agree there is expression of Y10b in the cytoplasm of the blastomeres. However, its absence from the nucleolus suggests reduction in rRNA intermediates more than likely due to the reduction in its synthesis, which is consistent with our specific cell type knockouts in post-implantation embryos.

Other comments

1. It’s known that there are both prenucleolar bodies that contain components of the rDNA transcription apparatus as well as bodies that also contain rDNA. The lack of nucleolar RNA (lack of Y10b staining, Figure 1), would suggest that the nucleolar “remnants” observed may be reflective of protein-protein interactions that are independent of rRNA synthesis/processing. Might these structures be similar to those observed immediately post-mitosis prior to the resumption of rDNA transcription?

- We agree with the reviewer that nucleolar remnants present in the Pol I mutant preimplantation embryos are probably protein-protein interactions and protein-DNA interactions (Figure 2D) in the absence of rRNA synthesis. However, these remnants cannot nucleate a functioning nucleolus unlike in post-mitosis cells.
- Regarding structure of the remnants, electron microscopy images of *Polr1c^-/-^* mutants are not comparable to late telophase or early G1 nucleoli imaged previously (Fig. 8 from Savino et al, JCB, 2001 and Fig. 3 from Fomproix et al, JCB, 1998), suggesting while the remnants are composed of protein and DNA, they are not structurally similar to post-mitosis cells.

2.Pol R1C and PolR1D are both subunits of Pol I and Pol III. Hence, the inclusion of those mutants does not enhance the primary conclusions of the manuscript. What happens if you knockout PolR1F?

- It would be interesting to know what happens in the absence of *Polr1f*, but that is beyond the scope of the current study. Generating and analyzing a new knockout mouse model is not trivial due to the cost and lengthy time needed, but it may also not provide substantially new information above and beyond the multiple Pol I specific subunit mouse mutants, and Pol I inhibited hiPSC models, already used in the study.
  - We generated four knockout mouse mutants, *Polr1a*, *Polr1b*, *Polr1c* and *Polr1d*. Polr1a and Polr1b are catalytic core subunits of Pol I while Polr1c and Polr1d are structural subunits of both Pol I and Pol III complexes. Considering our data from the knockout of each distinct subunit is consistent with respect to nucleolar structure and number, and that rRNA synthesis is significantly reduced irrespective of which subunit is deleted, this collectively suggests a role for the Pol I complex in rRNA transcription as well as in maintaining nucleolar structure.
  - However, it’s important to reiterate the inclusion of Polr1c and Polr1d in our studies because, despite being shared between Pol I and Pol III, unlike Pol I, Pol III is not a component of the nucleolus. Therefore, any changes observed in the nucleolus in the absence of Polr1c and Polr1d are indicative of their function in the Pol I complex.
  - Furthermore, it’s interesting to note that modeling Treacher Collins syndrome (TCS) pathogenic variants in *POLR1C in vitro* was shown to primarily affect Pol I function (Thiffault et al., 2015). Although another similar study performed in yeast revealed some TCS variants may affect both Pol I and Pol III (Walker-Kopp et al., 2017), this doesn’t change the fact that Pol III is not a component of the nucleolus.

3. The measurement of the intensity of staining of a particular protein might be indicative of its relocation from the nucleolus as reported in Supplemental Figure 1. However, it may also reflect its destruction as well as the redistribution of the protein throughout the nucleolplasm/cytoplasm.

- While we hypothesize that the reduction in protein intensity from the nucleolus of the Pol I mutants is due to its relocation to the nucleus, we agree that the data does not rule destruction as well as redistribution in the cytoplasm. We have therefore modified the language in the text to reflect this idea and provide a more balanced interpretation.

4.The authors miss the opportunity to determine if the knockout of a core Pol I subunit affects the steady-state levels of the other subunits as has been reported for the effects of BMH21.

- Identifying the steady state levels of Pol I subunits in the Pol I preimplantation mutants is a monumental task because antibodies against Pol I subunits are not yet available for mouse proteins. In addition, the number of Pol I preimplantation mutants required to perform the required experiments would be extremely large and almost prohibitive to collect.
- Irrespective of whether the other Pol I subunits are affected by the absence of Polr1a or Polr1b, the end result is a reduction in rRNA synthesis, which changes the phase separation properties of the nucleolus and consequently its structure and function. Therefore, the steady state levels of other subunits, which might be of biochemical interest, is likely not consequential to the data or model presented in this manuscript.

Reviewer #2: This study by Dash et al. investigates the impact of ribosomal RNA (rRNA) transcription on nucleolar structure in mammalian development. Transcription of rRNA, catalyzed by RNA Polymerase I (RNA Pol I), is the essential first step in ribosome biogenesis. Loss of function variants in RNA Pol I subunits cause congenital conditions, called ribosomopathies, that disproportionately impact craniofacial development. Previously, the authors showed that craniofacial differences in ribosomopathies result from impaired rRNA transcription, activation the P53-mediated nucleolar stress response, and selective apoptosis in neural crest cells. Since nucleolar stress is closely linked with changes in nucleolar structure, this study aims to determine how loss of RNA Pol I alters nucleolar organization and assembly during development. Herein they present compelling evidence that loss of RNA Pol I activity, either through genetic inactivation in mouse embryos or pharmacological inhibition in human iPSCs, disrupts liquid-liquid phase separation that is necessary to establish the nucleolus’ tripartite structure and promote nucleolar reassembly following mitosis. In the absence of rRNA transcription, liquid-liquid phase separation is disrupted due to increased viscosity of the nucleolus’ outer granular compartment (GC). Overall, this is a comprehensive study that sheds new light on a requirement for rRNA transcription on nucleolar structure and function in development and, potentially, the pathogenesis of ribosomopathies.

General Feedback:

1. To investigate potential changes in the nucleolar phase separation properties following RNA Pol I inhibition, FRAP was used to follow recovery of RFP-tagged NPM1. It is not clear why phase separation was examined only using RFP-NPM1 (which marks the GC and not also GFP-FBL (which marks the dense fibrillar component (DFC)). rRNA transcription occurs at the FC–DFC interface where Pol I complexes are enriched. Higher concentration of rRNA decreases FBL saturation concentration to induce preferential condensation of fibrillarin in the DFC and promote phase separation. Therefore, changes in the nucleolar phase separation properties (increased viscosity) upon RNA Pol I inhibition may be, in part, due to increased FBL.

- The reviewer raises an important point and indeed we attempted to perform FRAP on FBL, in addition to NPM1. However, due to the size and shape of the DFC, performing half and whole FRAP caused phototoxicity to the cells. We do agree with the reviewer, that upon Pol I inhibition we would expect to see changes in the viscosity of FBL as the concentration of rRNA decreases and the saturation concentration of FBL increases. Therefore, although we have focused on phase separation properties of the GC, we would expect phase separation changes to also occur in other compartments of the nucleolus including the DFC.

2. The models used here lead to complete loss of RNA Pol I activity (homozygous loss of function and pharmacological inhibition). There should be some discussion that addresses the following: How do these models shed light on ribosomopathies caused by partial loss of RNA Pol I activity? Is it expected that nucleolar morphology and assembly would be similarly impacted in cells where RNA Pol I activity is reduced but not completely lost?

- The reviewer raises an excellent question considering human patients have heterozygous mutations in Pol I subunits leading to Treacher Collins Syndrome and Acrofacial Dysostosis Cincinnati Type. In a recent publication from the Weaver and Trainor labs, Acrofacial Dysostosis – Cincinnati type disease associated POLR1A variants elicited reduced rRNA synthesis in concert with larger and fewer nucleoli, consistent with nucleolar dysfunction. Furthermore, a subset of variants rendered the nucleolar foci more extended and linear (Smallwood et al, 2023, ASHG Figure S2, S3, S4, please see figure below). This demonstrates that different variants may have distinct effects on nucleolar structure, but in each case the altered structure is still consistent with nucleolar dysfunction as part of developmental disorder pathogenesis.
- Moreover, we think this principle is likely to be widespread. For example, HMGB1 is an intrinsically disordered protein, and mutations in *HMGB1* cause brachyphalangy, polydactyly and tibial aplasia, a rare complex malformation syndrome. Recently disease causing variants were shown to alter HMGB1 phase separation, enhancing its partitioning into the nucleolus and causing nucleolar dysfunction in association with reduced rRNA transcription and nascent protein translation (Mensah et al, 2023, Nature).

- However, in the case of pre-implantation mouse embryos, heterozygous loss of any of the Pol I subunits does not seem to aberrantly effect prenucleolar bodies. We have added this data to the new Supplemental Figure 1.

3. How does this work reveal new insights into the phenotypic specificity for neural crest cells? For example, there is cell type-specific heterogeneity in the number of FC-DFC modules per nucleolus. Do the number of FC-DFC modules per nucleolus in neural crest cells make them more reactive to nucleolar stress in response to abnormal nucleolar structure?

- We have differentiated hiPSCs into neural crest cells (NCCs) *in vitro* and observed a dramatic reorganization of nucleolar structure (Figure below A, B). Compared to hiPSCs, the number of nucleoli per cell increases in differentiated NCCs, but the size per nucleolus decreases. These changes are consistent with published literature that suggests differentiation results in changes in nucleolar structure due to changes in chromatin structure. Additionally, when hiPSCs and NCCs are treated with the Pol I inhibitor, BMH-21, the NCCs undergo cell death after 24hrs of treatment, whereas the hiPSCs do not (Figure below C), suggesting the NCC are more sensitive to Pol I inhibition than hiPSCs. This is consistent with the threshold sensitivity of NCC to perturbations in Pol I function as demonstrated in our previous publication (Falcon et al., 2022 PNAS).
- However, this data is going to be included with a separate manuscript, which is in preparation and therefore, we have not added it to the current manuscript.

Minor edits:

1. Define “NORs” for the reader.

We have now defined nucleolar organizing regions (NORs) in the main text.

2. Please clarify the following sentence: The whole FRAP recovery (Tau2, Fig 3O) also increased with BMH-21 treatment, suggesting there is reduced affinity, or increased resistance to the addition of NPM1 to the GC underlying the increased recovery.

Sentenced clarified in manuscript.

3. Why is the level of Fbl expression variably affected in the Polr1 mutants? (Figure 1)

The reviewer raises another excellent question. We hypothesize that Fbl expression/localization is dependent on not only the quantity of rRNA present in the nucleolus but also on the other RNA-binding proteins and non-coding RNAs present in the nucleolus. For example, there is a correlation between Fibrillarin and Treacle expression. In Treacle antibody treated embryos, the levels of Treacle are reduced (Fig. S2 I-J), and the expression of Fibrillarin is correspondingly decreased as well. This trend is also observed in *Polr1d^-/-^* embryos where Treacle and Fibrillarin expression are the highest (Fig. 1D and F). However, since Fibrillarin interacts with a host of other RNA-binding proteins (Rodriguez-Corona, 2015, Biology of the cell) and long non-coding RNAs (Li et al, 2023, Nature Structural and Molecular Biology), its localization probably depends more broadly on the protein composition of the nucleolus.

4. That BMH-21 treatment of E8.5 embryos results in a single nucleolus and fragmented nucleoli phenotype could be more easily appreciated with higher magnification images of DAPI/Treacle/Pbl in Figure 3C and D.

We thank the review for this suggestion and have added higher magnification images to Fig 3 as Fig. 3 C’, D’ and D”.
